# The Principle of Least Action for Reversible Thermodynamic Processes and Cycles

**DOI:** 10.3390/e20070542

**Published:** 2018-07-21

**Authors:** Tian Zhao, Yu-Chao Hua, Zeng-Yuan Guo

**Affiliations:** Key Laboratory for Thermal Science and Power Engineering of Ministry of Education, Department of Engineering Mechanics, Tsinghua University, Beijing 100084, China

**Keywords:** principle of least action, optimization problems, reversible thermodynamic processes, Carnot cycle

## Abstract

The principle of least action, which is usually applied to natural phenomena, can also be used in optimization problems with manual intervention. Following a brief introduction to the brachistochrone problem in classical mechanics, the principle of least action was applied to the optimization of reversible thermodynamic processes and cycles in this study. Analyses indicated that the entropy variation per unit of heat exchanged is the mode of action for reversible heat absorption or heat release processes. Minimizing this action led to the optimization of heat absorption or heat release processes, and the corresponding optimal path was the first or second half of a Carnot cycle. Finally, the action of an entire reversible thermodynamic cycle was determined as the sum of the actions of the heat absorption and release processes. Minimizing this action led to a Carnot cycle. This implies that the Carnot cycle can also be derived using the principle of least action derived from the entropy concept.

## 1. Introduction

The principle of least action is a fundamental physics principle that is widely applied in physics [1]. In optics, Fermat proposed Fermat’s principle, which states that the path taken between two points by a ray of light is the path that can be traversed in the least time [2], such as a straight ray in a uniform medium, or the refraction of light passing through an interface between two media. In this principle, the traversal time is the optimization object and the product of the refraction index and the optical path is regarded as the action. After Fermat, Maupertuis and Euler independently proposed Maupertuis’s principle for mechanics, which states that the path followed by a physical system is the one having the shortest length (with a suitable interpretation of path and length). In this principle, the integral of the kinetic energy over time is the action, which is a special case of the more generally stated principle of least action [3]. Hamilton then proposed Hamilton’s principle in 1834, a general principle of least action for classical mechanics, which states that the dynamics of a physical system are determined by a variational problem for a functional based on its Lagrangian, which contains all the physical information concerning the system and the forces acting on it [4,5]. In irreversible thermodynamics, Onsager proposed the minimum energy dissipation principle, where the relationships for linear transport processes can be derived with the entropy production as the action of irreversible transport processes [6,7]. Prigogine developed the entropy generation minimization principle (EGMP), i.e., a steady state is characterized by the minimum of the entropy production function [8]. The principle of least action is also used in many other fields of physics, such as electrodynamics [9] and quantum mechanics [10]. Therefore, many researchers believe that the principle of least action is a basic physical law [11].

All these principles of least action can be applied to natural problems without manual intervention to derive the corresponding governing equations. Dharma-Wardana indicated that mechanical motion in nature already incorporates the principle of the least expensive route [12], which yields the notion that natural phenomena represent optimized processes. In this view, natural problems without manual intervention may be regarded as a category of optimization problems, with the action playing the role of the optimization criterion. Another class of optimization problem is that of one with outside intervention, such as the structure of a honeycomb, which is widely considered to be the optimal design for minimizing the amount of material used to reach the minimal weight and material cost [13]. Problems in this category all have optimization objects and artificial intervention. Similarly, actions for these optimization problems can also be taken as their optimization criteria, and their corresponding principles of least action can then be established. For instance, in the famous brachistochrone problem, the harmonic average velocity can be selected as the action to derive the optimal path [14]. For structure optimization problems in civil engineering, the total mass of the structure can be selected as the action to derive the optimal structure, i.e., the structure with the smallest weight satisfying all the design requirements [15]. For irreversible heat transfer problems, the entropy generation was taken as the action to optimize the heat transfer process by some researchers, which was called thermodynamic optimization [16,17,18,19,20,21]. However, Hua et al. [22] recently pointed out that the action of heat conduction should be entransy dissipation rather than entropy generation.

On the other hand, each discipline should have its own action, and its extreme value can lead to the fundamental law in that discipline. For example, the action in particle dynamics is the integral of the Lagrangian of the object or system with respect to the time interval [23], and Newton’s law of motion can be derived in terms of its variation. The action in optics is actually the optical path length of a ray [24], and the law of refraction can be derived in terms of its variation. The action in heat transfer is entransy dissipation (not entropy generation) [25], and Fourier’s law of heat conduction can be derived from its variation. That is why entransy dissipation, rather than entropy generation, is the optimization criterion of heat transfer without heat–work conversion. 

For both reversible thermodynamic processes and cycles, it seems that the concept of action and the principle of least action are seldom discussed in the literature. Therefore, this study analyzes a class of general reversible thermodynamic processes and cycles to develop their principle of least action, which may lead to the optimization of thermodynamic cycles.

## 2. The Brachistochrone Problem in Mechanics 

The optimization of a process usually refers to the search for a “path” with fixed initial and final states that corresponds to the optimization object. The brachistochrone problem in classical mechanics is a familiar optimization case, and is also very similar to the optimization of reversible thermodynamic processes; thus, we begin by briefly reviewing the brachistochrone problem. 

**Figure 1:**
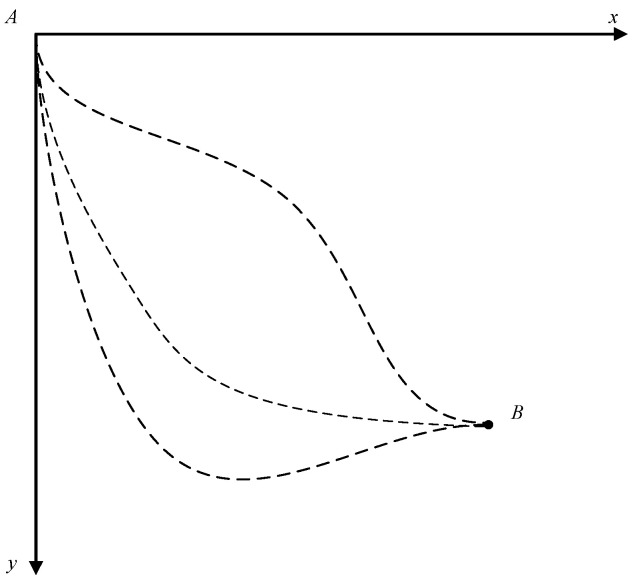
The brachistochrone problem in classical mechanics.

The problem can be stated as follows: suppose that a bead of mass *m* moves along a curved wire under the influence of gravity without friction, indicating a reversible motion. The particle moves, starting at rest, from fixed point *A* to fixed point *B*, as shown in Figure 1. The problem is to find the path function *y* = *y*(*x*) from point *A* to point *B* that minimizes the time integral:

(1) $$T=\int_{A}^{B}dt.$$

The classical method of solving this problem found in many textbooks expresses the time integral in an explicit form, before applying the Euler equation to obtain the optimal path, which is a cycloid [26]. However, this optimal path can also be obtained by using the Lagrange multiplier method as a functional, where the expended time, *T*, of the particle motion from fixed point *A* to fixed point *B* is the optimization object, and the harmonic average velocity is taken as its action. The minimum value of the action leads to the optimal path with the minimum expended time. That is, the principle of least action can also be applied to solve optimization problems once the action is properly chosen.

Since there is no friction, the mechanical energy in the system is conserved. If the zero of the potential energy is chosen as the *x*-axis, the potential energy is

(2) V(y)=mgy.

The principle of mechanical energy conservation yields

(3) $$T(y)+V(y)=-\frac{1}{2}mv^{2}+mgy=0,$$

where *T*(*y*) is the kinetic energy, and *V*(*y*) is the potential energy. This relationship is the system constraint, and the constructed Lagrange functional is

(4) $$J=\int_{A}^{B}dt+c\int_{A}^{B}\left(mgy-\frac{1}{2}mv^{2}\right)dt,$$

where the first term represents the time, and the Lagrange multiplier *c* is a constant, since the second term in the functional is an isoperimetric constraint. Next, the time element, d*t*, is replaced by the traversed distance element, d*s*, and Equation (4) turns into

(5) $$\begin{array}{c} J=\int_{A}^{B}\frac{ds}{v}+c\int_{A}^{B}\left(mgy-\frac{1}{2}mv^{2}\right)ds\\ =\int_{A}^{B}\left(\frac{1}{v}+c\left(mgy-\frac{1}{2}mv^{2}\right)\right)ds \end{array}.$$

Since the whole path length, *s*, is not fixed, the integral limits *A* and *B* need to be converted into the coordinates of the two fixed points. Taking the horizontal coordinate as an example,

(6) $$ds=\sqrt{1+{\left(\frac{dy}{dx}\right)}^{2}dx}.$$

Substituting this expression into Equation (5) gives

(7) $$J=\int_{0}^{x_{B}}\left(\frac{1}{v}+c\left(mgy-\frac{1}{2}mv^{2}\right)\right)\sqrt{1+{y^{\prime }}^{2}}dx.$$

The solution of the original problem, *y*(*x*), can then be deduced using variational calculus. Setting the variations of Equation (7) equal to zero yields

(8) $$v=\sqrt{2gy};$$

(9) $$\sqrt{2gy}\sqrt{1+{y^{\prime }}^{2}}=\frac{1}{c}.$$

Squaring both sides of Equation (9) and rearranging the terms gives

(10) $$\left(1+y{'}^{2}\right)y=1/\left(2gc^{2}\right)=k^{2},$$

where *k* is a non-negative constant related to the initial and boundary conditions. The solution of this differential equation can be represented by the following parametric equations:

(11) $$x=\frac{1}{2}k^{2}\left(\theta -\sin\theta \right),y=\frac{1}{2}k^{2}\left(1-\cos\theta \right).$$

**Figure 2:**
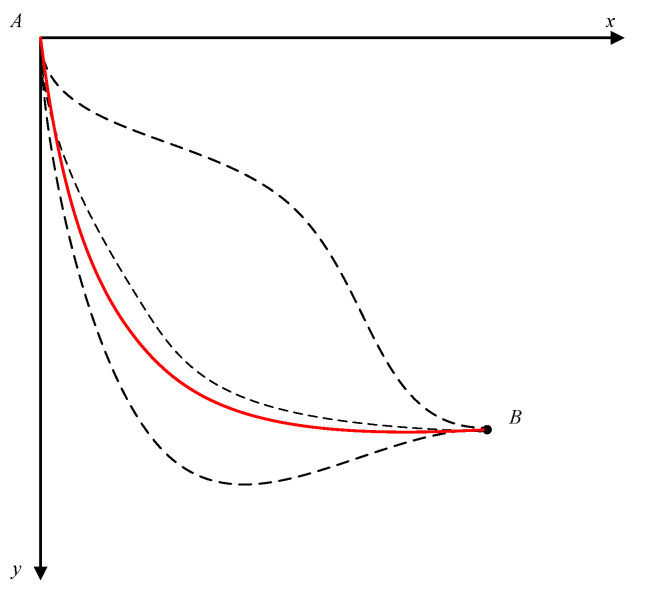
The solution of the brachistochrone problem.

Thus, the Lagrange multiplier method gives the same solution for the problem; the optimal path is a cycloid, as shown in Figure 2 by the solid line. Therefore, the brachistochrone problem is, in fact, the application of the principle of least action with the harmonic average velocity, $S_{AB}/\left(\int_{A}^{B}ds/v\right)$, as the action for particle motion in the earth’s gravitational field.

## 3. The Principle of Least Action and the Optimization of Reversible Thermodynamic Processes 

Since the purpose of a reversible heat absorption process is to convert the absorbed heat into work, the optimal process is one that maximizes the work output. According to the first law of thermodynamics, the work output is equal to the absorbed heat minus the increase in the internal energy of the system [27]:

(12) Q−ΔU=W.

Noting that the change in internal energy between two fixed states does not change within different paths, the heat absorbed into the system is equivalent to the work output in the perspective of optimization. Therefore, the optimal process can be defined as that which absorbs the maximum heat and outputs the maximum work.

The optimization of reversible thermodynamic processes is examined here based on the following assumptions: firstly, the system is a simple compressible thermodynamic system; secondly, the initial state, *A*, and final state, *B*, of the system are fixed; thirdly, heat is only absorbed during this process, which implies that *S_A_* ≤ *S* ≤ *S_B_*; finally, the system absorbs heat from a series of heat sources whose highest temperature is *T_A_*; thus, *T_B_* ≤ *T* ≤ *T_A_*. 

**Figure 3:**
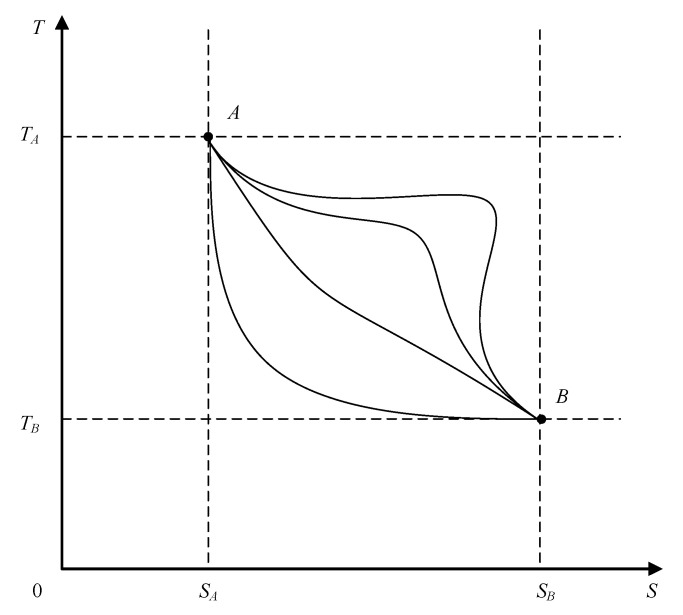
The thermodynamic system undergoing various reversible processes.

The *T–S* diagram for a reversible thermodynamic process, shown in Figure 3, presents several possible system paths between two state points (*A*, *B*) within the abovementioned temperature and entropy limits.

**Figure 4:**
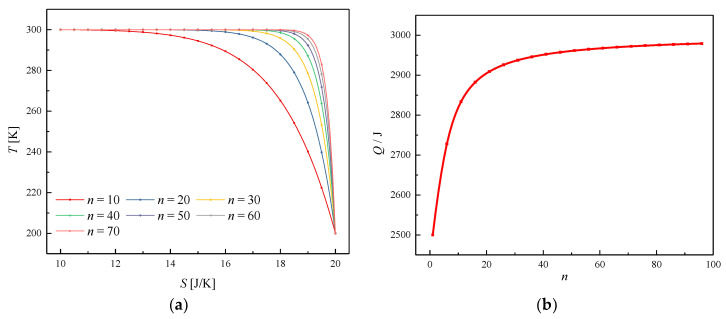
(**a**) *T*–*S* diagram for various paths with various function parameters. (**b**) The heat absorbed in the process versus the order of the power function, *n*.

Firstly, power functions with different orders (*n*) are used to qualitatively analyze the problem, as shown in Figure 4. Given that *T_A_* = 300 K, *T_B_* = 100 K, *S_A_* = 10 J/K, and *S_B_* = 200 J/K, Figure 4a presents several different power functions passing through points *A* and *B* in the *T–S* diagram as possible paths. Meanwhile, Figure 4b gives the heat absorbed within these thermodynamic processes. The power functions have the form, *T* = *C − DS^n^*, with each function having two constants, *C* and *D*, determined from the coordinates of *A* and *B*. As the order *n* increases, the power function tends to become a broken line, and the heat absorbed increases up to a limit, which gives guidance to the optimization process.

The action can be found for a reversible thermodynamic process, as with the brachistochrone problem. To determine which path absorbs the largest amount of heat, we need to relate the absorbed heat and the variation in fixed entropy [28].

(13) $$Q=\int_{A}^{B}TdS=\bar{T}\Delta S.$$

Equation (13) indicates that the largest amount of heat absorption requires the highest average temperature. That is, the system temperature in the process should be held as high as possible. Since the system temperature cannot exceed *T_A_*, the highest average temperature should be achieved through an isothermal process at *T_A_*:

(14) $$T=T_{\max}=T_{A}.$$

**Figure 5:**
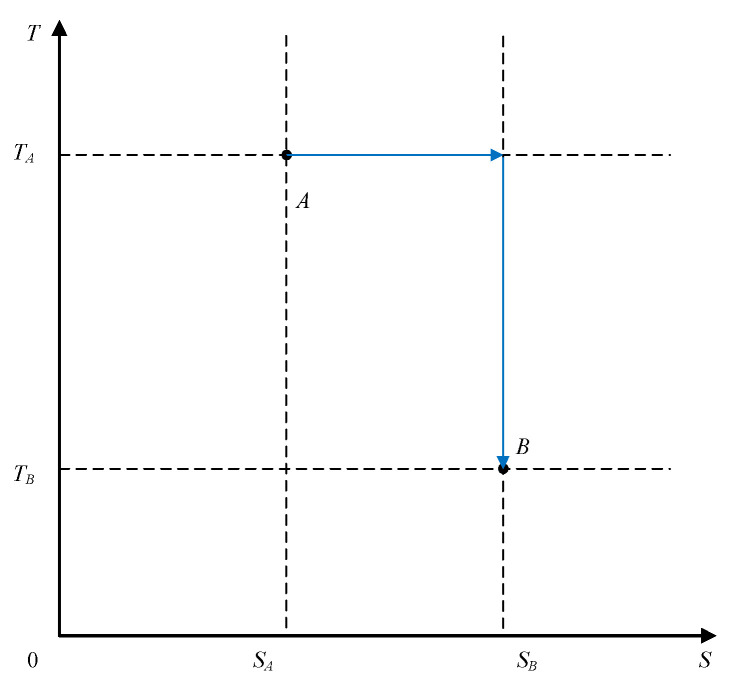
The optimal path maximizes the difference in heat between two fixed states.

However, the boundary condition requires that the temperature at the final state, point *B*, must be *T_B_*, which implies that the path with the largest amount of heat absorption is not smooth; rather, it has a Heaviside step function [29] at point *B*. As such, the temperature along the path is expressed by

(15) $$T=T_{A}+\left(T_{B}-T_{A}\right)H(S_{B}),$$

where *S_A_* ≤ *S* ≤ *S_B_*. Thus, the absorbed heat is maximized during the process from state *A* to state *B* when the system firstly undergoes an isothermal heat absorption process at *T_A_*, followed by an adiabatic expansion process, which is just a half of a Carnot cycle, as shown in Figure 5. Therefore, the quantity Δ*S_in_*/*Q_ab_*, i.e., the entropy increase per unit heat absorbed, is its action. The subscript *in* stands for increase (Δ*S_in_* > 0), and *ab* represents absorption (*Q_ab_* > 0).

**Figure 6:**
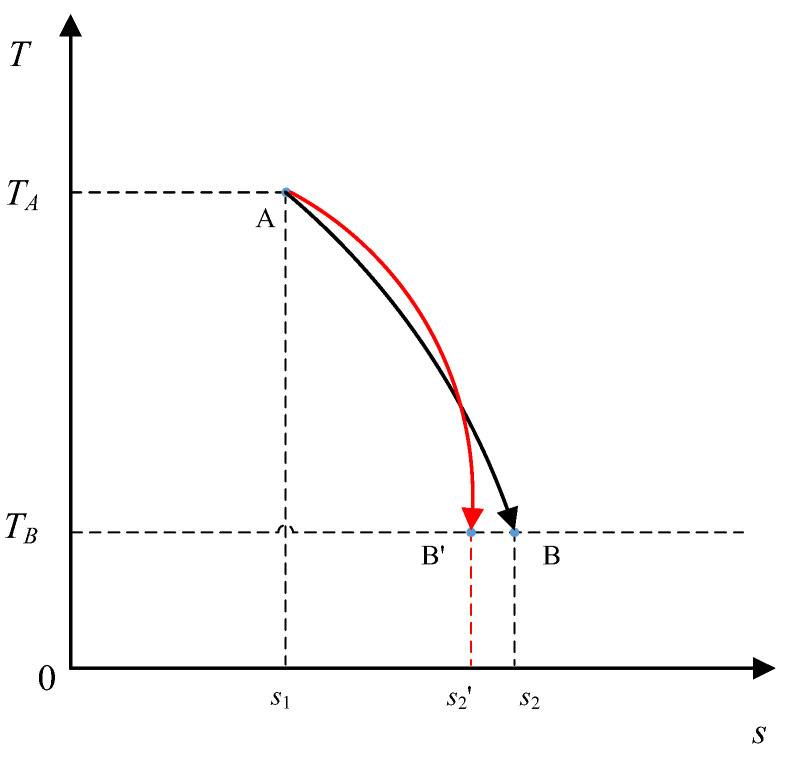
Two possible paths with a fixed initial state, a fixed temperature of the final state, and a fixed amount of absorbed heat.

Next, consider changing the second assumption such that the initial state *A* is still fixed; however, only the temperature of the final state of the system is fixed, as shown in Figure 6, along with the heat absorbed during the process also being fixed. The *T–S* diagram in Figure 6 shows two possible paths for the system between the initial state, point *A*, and the final state with temperature *T_B_* within the temperature limit.

In this case, the internal energy of the different final states at *T_B_* remains unchanged for the various reversible thermodynamic processes; thus, the fixed absorbed heat results in a fixed work output according to the first law of thermodynamics. However, the exergy and the entropy of the different final states will both differ depending on the final state. In this regard, the optimal process can be defined as the process that results in the minimum decrease in exergy or the minimum increase in entropy, while the absorbed heat is converted into the same amount of work.

The absorbed heat can be determined as

(16) $$Q=\int_{S_{A}}^{S_{B}}T(S)dS,$$

where *S* is the entropy corresponding to final state *B*. The path also has the endpoints

(17) $$T(S_{A})=T_{A},T(S_{B})=T_{B}.$$

Since the final state is not fixed, *S_B_* is not a fixed value. The average temperature for the whole process is defined as

(18) $$\bar{T}=\frac{\int_{S_{A}}^{S_{B}}T(S)dS}{S_{B}-S_{A}}=\frac{Q}{\Delta S}.$$

**Figure 7:**
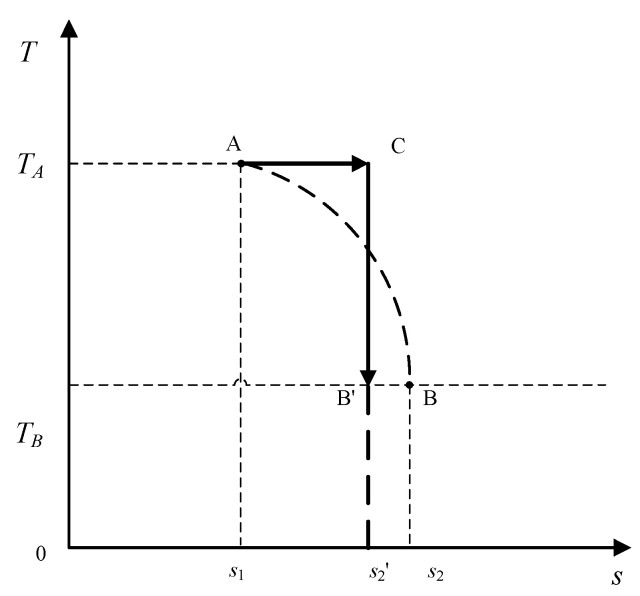
The optimal path with a fixed initial state, a fixed temperature of the final state, and a fixed amount of absorbed heat.

Equation (18) shows that the minimum increase in entropy requires the maximum average temperature during the thermodynamic process. Since the system temperature cannot exceed *T_A_*, the optimal process should be an isothermal heat absorption process at *T_A_*, followed by an adiabatic expansion process, which is, again, half of a Carnot cycle, as shown in Figure 7. Therefore, the entropy increase per unit heat absorbed, Δ*S*_in_/*Q*_ab_, is once again the action of the reversible thermodynamic process.

## 4. The Optimal Reversible Thermodynamic Cycle and Its Principle of Least Action

Any reversible thermodynamic cycle can always be divided into a heat absorption process and a heat release process. As noted earlier, the optimal heat absorption process consists of an isothermal heat absorption process, followed by an adiabatic expansion process. Similarly, the optimal heat release process can be shown to consist of an adiabatic compression process, followed by an isothermal heat release process, with the assumption that the heat is transferred to a series of heat sources, whose lowest temperature is *T_B_*, with *T_B_* ≤ *T* ≤ *T_A_*. The quantity Δ*S_de_*/*Q_re_*, i.e., the entropy decrease per unit heat released, is the action of the reversible heat release process. Here, the subscript *de* stands for decrease (Δ*S_de_* < 0), while *re* represents release (*Q_re_* < 0). The maximum value of Δ*S*_de_/*Q*_re_ yields the minimum heat release and the maximum exergy increase of the system for the fixed initial and final states.

**Figure 8:**
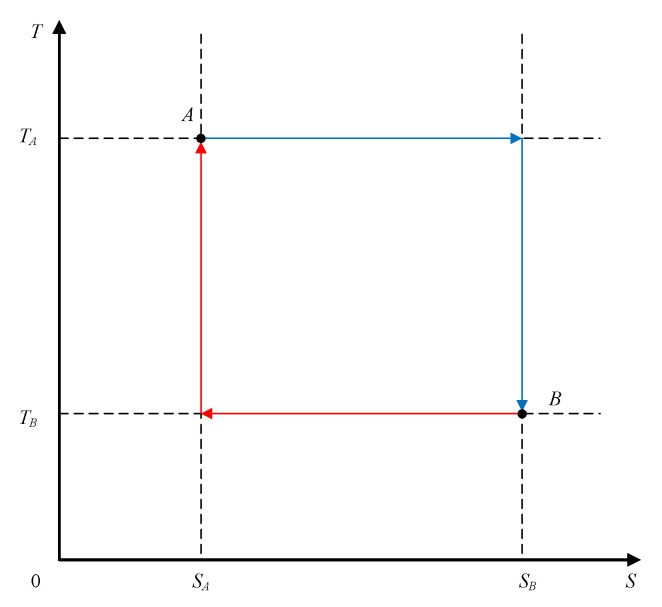
The optimal thermodynamic cycle between two fixed states.

Therefore, the optimal reversible thermodynamic cycle should be the combination of an optimal heat absorption process with ambient conditions as its fixed final state, and an optimal heat release process with ambient conditions as its fixed initial state, with two isothermal processes and two adiabatic processes, which simply results in a Carnot cycle, as shown in Figure 8.

Consequently, the action for a reversible thermodynamic cycle is the sum of the actions for the heat absorption process and heat release process: Δ*S_in_*/*Q_ab_* − Δ*S_de_*/*Q_re_*. Noting that $Q_{ab}={\bar{T}}_{ab}\Delta S_{in}$ and $Q_{re}={\bar{T}}_{re}\Delta S_{de}$, the action can be rewritten as $[\Delta S_{in}/Q_{ab}-\Delta S_{de}/Q_{re}]=1/{\bar{T}}_{ab}-1/{\bar{T}}_{re}$. That is, the optimum reversible thermodynamic cycle with a given temperature range (i.e., the Carnot cycle) can be derived based on the concept of entropy. This method might be considered a vicious circle, because the quantity known as entropy was introduced by Clausius, based on the Carnot cycle. However, as a state function of a thermodynamic system, the entropy can be derived without applying the knowledge of the Carnot cycle. For instance, Planck [30] and Caratheodory [31] both established the concept of entropy and the theorem of entropy while neither applying the Clausius equation nor the Carnot cycle. Hence, the principle of least action for a reversible thermodynamic cycle states that the minimum value of the action, Δ*S_in_*/*Q_ab_* − Δ*S_de_*/*Q_re_*, leads to the Carnot cycle, which is the optimal reversible thermodynamic cycle between two thermal reservoirs.

## 5. Conclusions

The principle of least action can be applied to two kinds of optimization problems for natural phenomena and for artificial phenomena. For natural processes, minimizing its action leads to its fundamental law, its governing relationship, and its real path, which is the optimal one corresponding to its optimization object. The principle of least action is not limited to reversible problems; rather, it is also applicable to irreversible problems such as heat conduction and convection. For artificial optimization problems, minimizing its action based on the known fundamental law and governing relationship leads to its optimal path, which corresponds to its optimization object. 

The optimization of reversible thermodynamic processes belongs to the artificial intervention class of optimization problems. The actions for heat absorption and heat release processes are the entropy variation per unit of exchanged heat, Δ*S*/*Q*. Minimizing this action leads to the minimum increase in entropy for a fixed absorbed heat during the heat absorption process, with the corresponding optimal path being the first half of a Carnot cycle. Maximizing this action leads to the minimum decrease in entropy for a fixed heat release during the heat absorption process, with the corresponding optimal path being the second half of a Carnot cycle.

The optimal reversible thermodynamic cycle is then the combination of the optimal reversible heat absorption process and the optimal reversible heat release process with ambient conditions. Therefore, the action of a reversible thermodynamic cycle is the algebraic sum of the actions of the heat absorption and heat release processes, (Δ*S*/*Q*)*_ab_* − (Δ*S*/*Q*)*_re_*. Minimizing this action leads to the optimal reversible thermodynamic cycle, i.e., the Carnot cycle. This shows that, like other disciplines, a variation of the action, (Δ*S*/*Q*)_ab_ − (Δ*S*/*Q*)_re_, may lead to the Carnot theorem, which is the fundamental law in reversible thermodynamics.
